# Use of the FDA nozzle model to illustrate validation techniques in computational fluid dynamics (CFD) simulations

**DOI:** 10.1371/journal.pone.0178749

**Published:** 2017-06-08

**Authors:** Prasanna Hariharan, Gavin A. D’Souza, Marc Horner, Tina M. Morrison, Richard A. Malinauskas, Matthew R. Myers

**Affiliations:** 1 US Food and Drug Administration, Silver Spring, Maryland, United States of America; 2 ANSYS, Inc., Evanston, Illinois, United States of America; University at Buffalo - The State University of New York, UNITED STATES

## Abstract

A “credible” computational fluid dynamics (CFD) model has the potential to provide a meaningful evaluation of safety in medical devices. One major challenge in establishing “model credibility” is to determine the required degree of similarity between the model and experimental results for the model to be considered sufficiently validated. This study proposes a “threshold-based” validation approach that provides a well-defined acceptance criteria, which is a function of how close the simulation and experimental results are to the safety threshold, for establishing the model validity. The validation criteria developed following the threshold approach is not only a function of Comparison Error, E (which is the difference between experiments and simulations) but also takes in to account the risk to patient safety because of E. The method is applicable for scenarios in which a safety threshold can be clearly defined (e.g., the viscous shear-stress threshold for hemolysis in blood contacting devices). The applicability of the new validation approach was tested on the FDA nozzle geometry. The context of use (COU) was to evaluate if the instantaneous viscous shear stress in the nozzle geometry at Reynolds numbers (Re) of 3500 and 6500 was below the commonly accepted threshold for hemolysis. The CFD results (“S”) of velocity and viscous shear stress were compared with inter-laboratory experimental measurements (“D”). The uncertainties in the CFD and experimental results due to input parameter uncertainties were quantified following the ASME V&V 20 standard. The CFD models for both Re = 3500 and 6500 could not be sufficiently validated by performing a direct comparison between CFD and experimental results using the Student’s t-test. However, following the threshold-based approach, a Student’s t-test comparing |S-D| and |Threshold-S| showed that relative to the threshold, the CFD and experimental datasets for Re = 3500 were statistically similar and the model could be considered sufficiently validated for the COU. However, for Re = 6500, at certain locations where the shear stress is close the hemolysis threshold, the CFD model could not be considered sufficiently validated for the COU. Our analysis showed that the model could be sufficiently validated either by reducing the uncertainties in experiments, simulations, and the threshold or by increasing the sample size for the experiments and simulations. The threshold approach can be applied to all types of computational models and provides an objective way of determining model credibility and for evaluating medical devices.

## Introduction

In spite of the wide adoption of computational fluid dynamics (CFD) in medical device design and research, these tools have not realized their full potential in the regulatory process [1]. In general, the influence of modeling and simulation on regulatory decision-making has been fairly minimal, with companies relying primarily on bench testing, animal testing, and clinical trials as evidence of device safety [2]. One of the main reasons for the limited use of CFD (and other physics-based simulations) is the challenge of adequately establishing credibility for making safety claims.

The key aspects for establishing model credibility are to perform appropriate verification, validation, and uncertainty quantification (VVUQ) to ensure that the model results are sufficiently accurate for the prescribed context of use (COU), which refers to the specific role and scope of the computational model and the simulation results used to inform a decision [3]. Good VVUQ practices are prevalent and well-documented in other engineering fields such as the aerospace and automotive industries [4–11]. Sandia National Laboratories and other research groups have also published reports on VVUQ approaches for various applications [12, 13]. Additionally, the American Society of Mechanical Engineers (ASME) has a standards committee devoted to verification and validation (V&V); they have published two standards, V&V 10–2006 and V&V 20–2009, that outline steps for performing VVUQ in solid and fluid mechanics, respectively [14, 15].

Researchers in the biomedical industry have begun to perform rigorous validation of their CFD models and codes by comparing with *in vitro* [16–19], *in vivo* [20, 21] or clinical data [22, 23]. In recent years, few researchers have applied VVUQ techniques in the development of more credible CFD models. Sankaran et al. [24] described a stochastic collection method for uncertainty quantification in cardiovascular simulations. They applied the stochastic method to samples problems including an abdominal aortic aneurysm and idealized and patient-specific Fontan surgery problems. Schiavazzi et al. [25] used uncertainty quantification approach for predicting the clinical outcome during single ventricle palliation surgery. The input parameters for their CFD model, such as the pulmonary pressure and flow split ratio, and their associated uncertainties were determined from preoperative clinical data using an inverse problem. The input parameter uncertainties were than propagated to the CFD output (e.g. pressure, velocity and wall shear stress) using sparse grid stochastic collection. Tran et al. [26] used a similar Monte Carlo sampling method to study coronary artery stenosis and quantified patient-specific uncertainties in hemodynamic parameters such as time averaged wall shear stress (TAWSS) and oscillatory shear index (OSI) based on fluctuations in the non-invasively measured clinical input data. Similar types of uncertainty quantification methods have been used by a few other research groups for cardiovascular and intracranial aneurysm modeling [27–29].

While the application of rigorous VVUQ methods is beginning to receive attention in the biomedical industry, the application of these methods in regulatory submissions is not widespread. The medical device community including academia, industry, the FDA and software developers, has been pursuing efforts to improve and standardize the process for establishing model credibility requirements. Several groups have sponsored round-robin studies to evaluate the ability of commercial and open-source CFD codes to accurately simulate fluid flow in various benchmark medical devices and physiological geometries [30–34]. These benchmark geometries and the corresponding validation data are increasingly being used by modelers to test their new and existing CFD codes [35–40]. Additionally, FDA has recently published a guidance document that provides detailed methodology on how to report computational modeling studies as a part of regulatory submissions [41]. Finally, FDA and industry are collaborating on the development of a risk-informed credibility assessment framework through the ASME V&V 40 subcommittee, which is devoted to verification and validation of computational modeling as applied to medical devices [3].

The most common type of validation approach seen in the medical device literature is the comparison of mean data from validation experiments with a single representative simulation (which aims to mimic the mean experimental conditions). Typically, the comparison between these data is qualitative and statements like “reasonable agreement” are used to describe the comparison. This approach does not provide sufficient validation for a majority of use cases because variation and uncertainty in the input parameters, such as device geometry, fluid properties, and inlet and outlet boundary conditions, have not been accounted for but are crucial for simulating the validation experiments. Depending on the problem, many, if not all, of these input parameters will have uncertainties which can significantly affect the model output(s). Similarly, there are uncertainties associated with the equipment and procedures used in the experiments that provide the validation data [14], [42]. Consequently, in addition to comparing the mean data from experiments and simulations, a quantitative estimation of all the uncertainties is also required to determine the level of confidence with which the difference between the experiment and computation can be estimated.

Even in the presence of rigorous quantification of the relevant uncertainties, a decision on whether a model is sufficiently validated is often subjective. The process of establishing the credibility of computational models awaits both improved adherence to rigorous error quantification, and the development of objective mechanisms for determining that the model has satisfied the validation criteria for a specific COU. For example, an objective evaluation of the validation criteria should take into account the model risk. Model risk, as defined by ASME V & V 40 standard [3], is the possibility that the computational model may lead to an incorrect decision that might result in patient harm or undesirable non-patient related impacts. The validation criteria, i.e., allowable difference between the computational model and experimental results should be dependent on the model risk.

As an example, the COU for the computational model in this study is to predict the instantaneous viscous shear stress and determine if it is low enough to avoid red blood cell damage (hemolysis) in the nozzle device (a flow channel of variable diameter) for throat Reynolds numbers of 3500 and 6500 (Table 1). For this COU, the validation criterion, which is the acceptable difference between the experimental and the CFD shear stresses, can be determined by assessing the magnitude of the difference between CFD and experimental data as compared to the difference between CFD and the hemolysis threshold. Previous experimental studies have reported threshold viscous shear-stress values for causing instantaneous hemolysis in medical devices [43]. If the CFD and experimental shear values are far from the threshold, the model risk is low and a less stringent validation criterion can be used for validating the CFD results. As the CFD and experimental shear stresses get closer to the threshold shear stress, the model risk increases, and a more stringent validation criterion needs to be applied for comparing the CFD model to the experiment. In order to account for model risk in the validation process, there is a need to use a threshold-based approach, in which the validation criteria is non-arbitrary and is determined by how close/far the CFD results are from the safety threshold.

**Table 1:** Context of use.

**Use CFD modeling to predict the instantaneous viscous shear stress and determine if it is low enough to avoid red blood cell damage in the nozzle device for throat Reynolds numbers of 3500 and 6500**

This paper describes a study in which bench tests and computational analyses of the FDA nozzle model [30] are used to illustrate techniques for rigorous error quantification. Additionally, we introduce a threshold-based method for determining that the model is sufficiently validated for the COU. For the prescribed COU (Table 1), techniques in the ASME V&V 20–2009 standard are applied to estimate i) the comparison error (E = |S-D|), which represents the difference between the mean experiment (D) and simulation (S) results, and ii) the validation uncertainty (U_val_), which is a summation of the uncertainties in the numerical simulations, input parameters and the experimental data. Our study then takes the analysis one step further by computing a test statistic based upon the uncertainties in shear-stress measurements and computations, and the assumptions of a two-tailed t-test. The statistic is used to compute a confidence interval for the difference between computations and measurements. Comparing the width of this confidence interval to the difference between the computational shear stress and the available thresholds for red blood cell damage (due to viscous shear) permits an objective determination of the credibility of the computational model for predicting the potential for blood-damage.

The COU for this study only covers the viscous shear stress and does not include the risk of hemolysis due to turbulent stresses [44]. Consequently, the viscous shear threshold used in this study (and hence the COU) is not applicable for scenarios where turbulence is expected to play a major role in causing blood damage in the device.

A brief outline of this paper is as follows. The details of the flow geometry and CFD solver are provided in the Methods section along with the steps involved in quantifying the validation uncertainty as described in the ASME V&V 20–2009 standard. Results from the uncertainty analysis and the subsequent discussion on threshold-based validation are provided in the Results and Discussion sections, respectively.

## Methods

Details of the computational model, numerical framework and the uncertainty quantification procedure are provided in this section.

### Nozzle model

**Fig 1:**
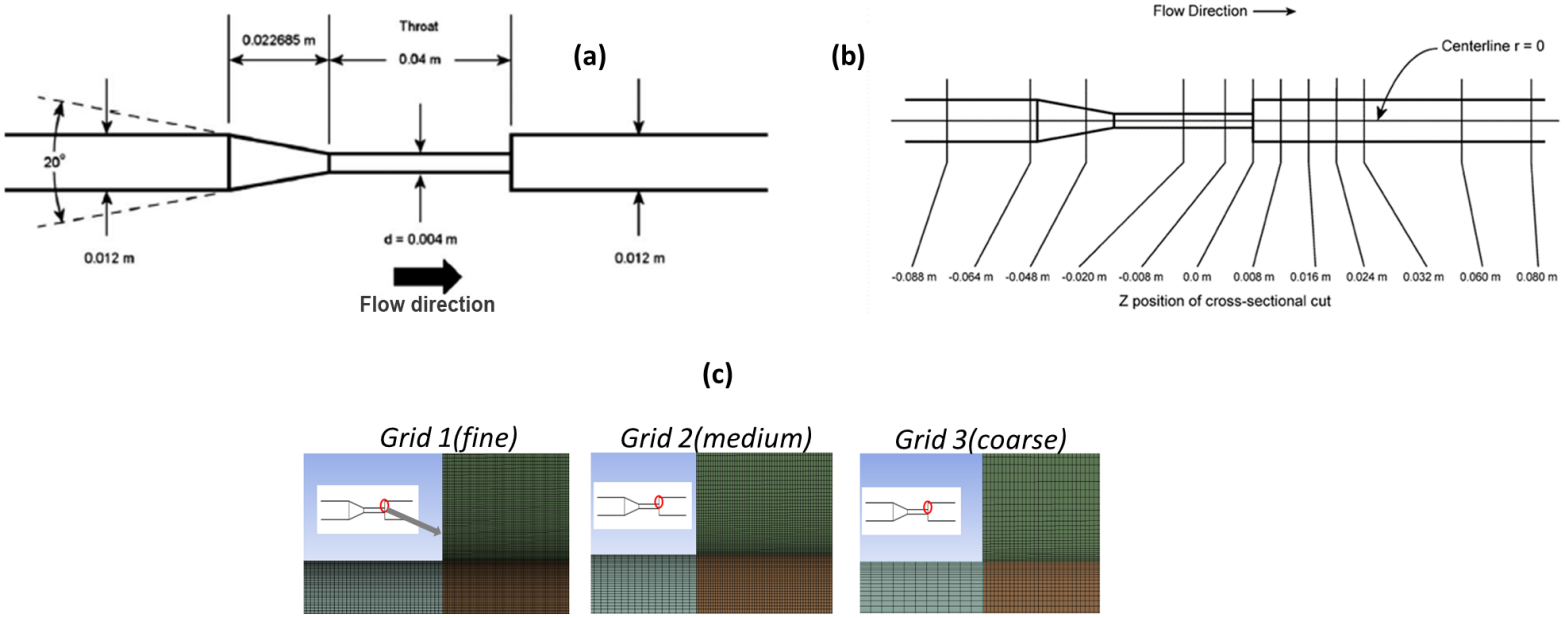
a) Schematic of the FDA nozzle model b) Cross-sectional cuts identify validation locations along the nozzle axis c) An image of the three grids (inside the red oval region) used for the grid convergence study (snapshot taken at the sudden expansion location).

The FDA benchmark nozzle model represents a simplified medical device; it is shown in Fig 1a and 1b with all relevant dimensions. The details regarding the nozzle geometry are provided in prior publications [30, 31, 33]. The nozzle has characteristics similar to blood contacting devices such as catheters, cannulae, and syringes. Geometrically, the model includes a gradually converging section, a throat region and a sharp-edged sudden expansion. These geometric features induce complex flow phenomena including adverse pressure gradients, recirculating flow and high and low shear stress zones, which mimic flow features of cardiovascular devices such as those found in the diffuser regions of blood pumps and ventricular assist devices. Such phenomena have been commonly linked to the occurrence of hemolysis and thrombosis in blood contacting devices [30].

### Computational model overview

A 3D geometric model of the nozzle was created using the commercial geometry creation software package (DesignModeler v.15.0, ANSYS, Inc., Canonsburg, PA). Assuming axisymmetric flow through the nozzle i.e., no variation in flow parameters in the circumferential (theta) direction, the numerical computations were performed for a 1° sector of the nozzle. The domain was discretized into finite volumes represented by hexahedral elements (Fig 1c) using the ANSYS Meshing Platform v.15.0 (ANSYS, Inc., Canonsburg, PA). Mesh density was increased in regions exhibiting complex flow dynamics such as high velocity gradients, adverse pressure gradients, separated flow, jet breakdown, and flow reattachment. The grid adjacent to the nozzle wall was refined by adding an inflation layer consisting of a higher number of hexahedral elements in comparison to the nozzle core. A grid convergence study was conducted in order to obtain a grid-independent solution, minimize the numerical error, and estimate the numerical uncertainty due to finite volume discretization.

### Input parameters

Input parameters for the CFD simulations were determined from an inter-laboratory particle image velocimetry (PIV) study, consisting of 5 datasets from 3 laboratories. This study analyzed the flow patterns of a blood analog solution flowing through the FDA nozzle [30]. Mean values and the uncertainty bounds for the inlet/outlet boundary conditions are summarized in Table 2. The blood analog solution was assumed to behave as a Newtonian fluid with a mean density,ρ, of 1056 kg/m^3^ and a mean dynamic viscosity, μ, of 0.0035 Pa-s. A mean Reynolds number of Re (= (4*ρ*Q)/(π*d*μ)) = 3500 or 6500 was used in this study, where *Q* is the inlet flow rate and *d* is the nozzle throat diameter (4 mm). A parabolic axial velocity profile (Eq 1), representing a fully-developed steady pipe flow condition, was imposed at the inlet boundary of the nozzle.

$$U_{z}\left(r\right)=2.{\bar{U}}_{z}\left[1-{\left(\frac{r}{R}\right)}^{2}\right]$$ (1)

where R is the nozzle inlet radius and ${\bar{U}}_{z}$ is the average axial velocity calculated from flow rate, *Q*. The turbulent intensity, *TI*, obtained from the PIV experiments, was also specified at the inlet. A stress-free boundary condition was applied at the nozzle outlet boundary. The nozzle wall was modeled using a no-slip velocity condition. A symmetry condition was applied to the two lateral (side) surfaces of the sector geometry model. Steady-state numerical computations were conducted using the finite-volume commercial CFD solver CFX v. 150.0 (ANSYS, Inc., Canonsburg, PA). Based on the Reynolds number of 3500 and 6500, a shear-stress transport (SST) k-ω turbulence model was used for solving the mass and momentum conservation equations. The advection terms in the discretized conservation equations were solved using a second-order accurate scheme. The convergence criteria for the residuals of the mass and momentum conservation equations were set to 1e-06. The simulations were conducted using a Dell T7400 workstation (4 GB RAM, 500 GB Hard drive) with a Windows 7 64-bit OS and a 2.49 GHz dual-core processor. Numerical uncertainty (*U*_*num*_) was estimated from the results of a mesh convergence study consisting of a series of three computational meshes (shown in Fig 1c). Grid #1 was used for obtaining the final results and conducting an input parameter uncertainty analysis.

**Table 2:** Values of the input parameters used for the input parameter uncertainty analysis.

Input parameter (*X*)	Throat Re~ 3500		Throat Re~ 6500	
	Nominal value	*uncertainty* (%)	Nominal value	*uncertainty* (%)
Volumetric flow rate, *Q*	3.59E-05ml/min	6.53%	6.89E-05 ml/min	7.86%
Dynamic viscosity, *μ*	3.50E-03 Pa.s	5.00	3.50E-03 Pa.s	5.00
Turbulent Intensity, *TI*	1.6%	65.07	5.45%	85.84%

### Estimation of uncertainties

The uncertainties in the output quantities of interest (i.e. velocity and shear stress) due to the numerical error associated with the discretization process, and uncertainty in the input parameters, were estimated using the following methodology [14].

#### i) Numerical uncertainty (U_num_)

The Grid Convergence Index (GCI) method [14], based on Richardson extrapolation theory, was adopted for conducting the grid convergence study and for obtaining the numerical uncertainty due to discretization of the flow domain into finite volumes. Firstly, a representative grid size was defined as $h={\left[\left(\sum_{i=1}^{N}\Delta V_{i}\right)/N\right]}^{1/3}$, where N is the total number of grid elements and Δ*V*_*i*_ the volume of the *i*^th^ grid element. Subsequently, a grid refinement factor was defined as *w*_21_ = *h*_2_/*h*_1_, where *h*_2_ and *h*_1_ are grid sizes that result in medium-density and high-density grids, respectively. Three grid sizes were chosen for the grid convergence study (grid #3—coarse, grid #2—medium, grid #1—fine) and were generated using a constant *w* value (i.e. *w*_21_ = *w*_32_ = *w*) of 2. The details of the three grids, including the total number of grid elements and nodes, are provided in Table 3.

**Table 3:** Details of the grids used for the grid convergence study.

Grid #	Refinement factor	Number of elements	Number of nodes
1 (fine)	-	486600	979636
2 (medium)	1.5	134700	272648
3 (coarse)	1.5	35250	72132

The axial velocity, *U*_*z*_, at the nozzle centerline and the wall shear stress, *τ*_*w*_, were selected as output variables of interest for assessing grid convergence. The centerline *U*_*z*_ was monitored at three axial locations, z = 0.0 m, z = 0.008 m and z = 0.06 m (Fig 1b) and *τ*_*w*_ was monitored at two axial locations, z = -0.04 m and z = -0.02 m, the latter corresponding to locations where the shear stress was assumed to be maximum. Finally, the numerical uncertainty was estimated using the GCI formula [14]:

$$U_{num}=GCI_{fine}^{21}=\frac{F_{s}\times \left|\frac{\varphi_{2}-\varphi_{1}}{\varphi_{1}}\right|}{w^{\theta }-1}$$ (2)

where *θ* = [1/ln (*w*)][*ln*|*ε*_32_/*ε*_21_|], and *ε* is the absolute error in the output variable of interest, *φ*, which was computed using two subsequent grids (for example, grids #1 & #2), e.g., *ε*_21_ = *φ*_2_−*φ*_1_. A factor of safety, *F*_*s*_, value of 1.25 was selected based on previous empirical studies for a three-grid convergence study [14].

#### ii) Input parameter uncertainty analysis

The uncertainty in the quantities of interest (i.e. simulation outputs) due to uncertainty in the simulation input parameters was estimated using the sensitivity coefficient method for uncertainty propagation [14]. This method is a local approach which assesses the effect of a small perturbation in the input parameter(s) on each simulation output parameter. While not performed here, the ASME V&V20-2009 recommends using Monte Carlo analysis to provide a global estimate of the input parameter uncertainty [14]. From the experimental measurements, volumetric flow rate, *Q*, dynamic viscosity, *μ*, and turbulent intensity, *TI*, were selected as the input parameters for the uncertainty analysis (Table 2). Inter-laboratory variability in other input parameters, such as the device dimensions and the fluid density, were not significant enough to warrant their inclusion in the uncertainty analysis. The output quantities of interest are identified based on the COU of the computational model. The quantities of interest for the COU of this analysis are axial velocity, *U*_*z*_, and shear stress, *τ*, which are the primary variables used to predict hemolysis potential. For each case, the overall uncertainty in each simulation output parameter due to uncertainty in the input parameters was calculated using Eq 3.

$$U_{inp}^{2}=\sum_{i=1}^{3}{\left(\frac{\partial S}{\partial X_{i}}u_{x_{i}}\right)}^{2}$$ (3)

where *S* represents an output parameter, *X*_*i*_ represents the *i*^*th*^ input parameter, $\frac{\partial S}{\partial X_{i}}$ is the sensitivity coefficient for output parameter *S*, and *$u_{x_{i}}$* is the experimental uncertainty in the *i*^*th*^ input parameter. The sensitivity coefficient was obtained using a second-order accurate central difference Taylor series expansion, $\frac{\partial S}{\partial X_{i}}=\frac{S\left(X_{1},\;\;\;X_{2},\ldots ,\;X_{i}+\Delta X_{i},\ldots ,\;X_{n}\right)-S\left(X_{1},\;\;\;X_{2},\ldots ,\;X_{i}-\Delta X_{i},\ldots ,\;X_{n}\right)}{2\Delta X_{i}}+O\left(\Delta X_{i}{}^{2}\right)$. Each input parameter (*X*_*i*_) was perturbed above and below its nominal (mean) value by a relative perturbation size, *ΔX/X*, of 7.5e-04. This perturbation size was chosen in order to minimize the truncation and round-off errors [14]. The uncertainty bounds for the input parameters were obtained from the experimental PIV study and are tabulated in Table 2. A total of seven simulations (2*n*+1, where *n* = 3 is the number of input parameters considered in the sensitivity study) were performed. In order to conduct a comprehensive uncertainty analysis that spanned the entire length and radius of the nozzle, *U*_*inp*_ for each simulation output parameter was estimated at multiple locations (Fig 1b). All results were analyzed using CFD-Post v. 15.0 (ANSYS, Inc., Canonsburg, PA) and custom developed codes (Matlab, *MathWorks*).

#### iii) Experimental uncertainties (U_exp_)

The velocity and shear stress measurements were collected at three different laboratories with the measurements repeated thrice at one laboratory. Therefore, a total of 5 datasets were available for calculating the mean and standard deviation of the experimental data. The experimental uncertainty was estimated as the standard deviation in the measurement data from these 5 datasets.

#### iv) Validation uncertainty (U_val_)

The validation uncertainty combines the uncertainties due to numerical errors (U_num_), input parameters (U_inp_), and experimental measurements (U_exp_) and is calculated as

$$U_{val}=\sqrt{U_{num}^{2}+U_{inp}^{2}+U_{exp}^{2}}$$ (4)

As per ASME’s V&V 20–2009 standard, U_val_ provides uncertainty limits for the comparison error, E, which is the difference between the mean experimental and simulation results. U_num_ (due to discretization error) was calculated at select locations. However, U_num_ is negligible (<1%) at the region of peak shear-stress when compared to input parameters and experimental uncertainties. The modeling error (*δ*_*model*_), which is the error in the simulation due to modeling assumptions and approximations, is bounded using U_val_ as follows

$$\delta_{model}\;\epsilon \;\left[E-U_{val},\;E+U_{val}\right]$$ (5)

Additionally, the comparison between U_val_ and E provides a metric for deciding whether the validation data can be used for reducing the model-form error and improving the model accuracy. If *U*_*val*_ << *E*, then *δ*_*model*_ ≈ *E* and the validation data can be used to guide changes to the model and minimize the comparison error to an acceptable level. On the contrary, if *U*_*val*_ >> *E*, then *δ*_*model*_ is of the order of *U*_*val*_, which means the noise level in the comparison error is too large to guide improvements to the computational model without first minimizing the various uncertainties.

### Threshold-based validation approach

In addition to calculating *U*_*val*_, we developed an alternate approach for establishing validation criteria which accounts for the COU of the computational model and is applicable for situations where threshold values for safety are available. For the COU in this paper, which is to evaluate the potential for hemolysis due to viscous shear stress in the flow nozzle, there are widely accepted threshold values available that relate viscous shear stress and hemolysis [43], [45]. For situations where threshold values for safety are available, our hypothesis is that a model can be considered “sufficiently validated” if the difference between the simulation results and validation experiments (defined as the comparison error, E, in this study) is less than the difference between the simulation results and the threshold. Accordingly, the allowable value for *E* is a function of how close the simulation results are to the safety threshold. As the simulation results approach the value of the safety threshold, the allowable limit for E is refined, so as to ensure that the model is sufficiently validated.

To present the threshold approach mathematically, we begin by defining the “proximity” *Ω* as the magnitude of the difference between the simulation result and the threshold: *Ω* = *|Threshold-S|*. If the uncertainties in the experiments, CFD model and threshold are properly quantified, then both *E* and *Ω* can be expressed in terms of confidence intervals as *CI*_*E*_ and *CI*_*Ω*_. We propose that a model can be considered sufficiently validated if *E* is less than *Ω* and *CI*_*E*_ and *CI*_*Ω*_ are non-overlapping (max(*CI*_*E*_) <min(*CI*_*Ω*_)). The limits of the confidence intervals for *E* and *Ω* can be determined using the expression for the difference of two normally distributed random variables with unequal variance. The confidence interval CI_Ω_ for Ω is given by [46]:

$$CI_{\Omega }=[\Omega -t_{d_{1}^{"},1-\alpha /2}\sqrt{B_{1}^{2}/n_{1}+B_{2}^{2}/n_{2}},\;\Omega +t_{d_{1}^{"},1-\alpha /2}\sqrt{B_{1}^{2}/n_{1}+B_{2}^{2}/n_{2}}\;]$$ (6)

where t is critical values of the Student’s t-distribution [46], the degrees of freedom $d_{1}^{"}=\frac{{\left(B_{1}^{2}/n_{1}+B_{2}^{2}/n_{2}\right)}^{2}}{{\left(B_{1}^{2}/n_{1}\right)}^{2}/\left(n_{1}-1\right)+{\left(B_{2}^{2}/n_{2}\right)}^{2}/\left(n_{2}-1\right)}$, *n*_*1*_ and *n*_*2*_ are the number of samples associated with each variable (*n*_*1*_ = 5 and *n*_*2*_ = 2 to 4 in this case), α = 0.05 (for 95% CI limit), B_1_^2^ is $U_{sim}^{2}=\;U_{num}^{2}+U_{inp}^{2}$ and B_2_^2^ is $U_{th}^{2}$, with *U*_*th*_ being the uncertainty in the threshold. An analogous expression holds for *CI*_*E*_ and can be written as

$$CI_{E}=\left[\text{ E}-t_{d_{2}^{"},1-\frac{\alpha }{2}}\sqrt{B_{1}^{2}/n_{1}+B_{3}^{2}/n_{2}},\text{ E}+t_{d_{2}^{"},1-\alpha /2}\sqrt{B_{1}^{2}/n_{1}+B_{3}^{2}/n_{2}}\;\right]$$ (7)

where the degrees of freedom, $d_{2}^{"}=\frac{{\left(S_{1}^{2}/n_{1}+S_{3}^{2}/n_{3}\right)}^{2}}{{\left(S_{1}^{2}/n_{1}\right)}^{2}/\left(n_{1}-1\right)+{\left(S_{3}^{2}/n_{3}\right)}^{2}/\left(n_{3}-1\right)}$, For the current example n_1_ = n_3_ = 5 (number of trials), B_1_ and B_3_ are the variances for the CFD (U_sim_) and PIV (U_exp_) datasets.

**Fig 2:**
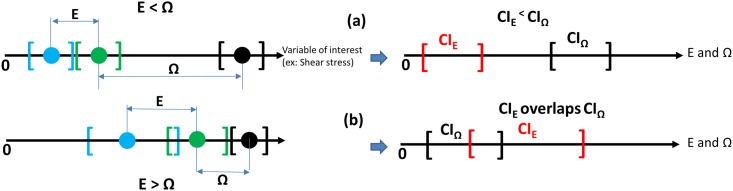
Qualitative explanation of the threshold approach for two different scenarios Black—Threshold; Green—CFD simulation and Blue—Experiment. [] represent mean±uncertainty. a) Model is valid and b) Model not necessarily valid. In scenario (a), both the experiments and simulations are far away from the threshold and CI_E_ smaller than CI_Ω_. In scenario (b), the simulations are closer to the threshold than to the experiments and CI_E_ is larger than CI_Ω_.

Fig 2 illustrates the threshold-based validation approach qualitatively for two different scenarios. In the first scenario (Fig 2a), the uncertainties for CFD and experiments (represented by green and blue square brackets, respectively) are non-overlapping. However, E is less than Ω and the CI_E_ and CI_Ω_ are non-overlapping suggesting that the computations still provide valid data for shear stress. In the second scenario (Fig 2b), the CFD and the experimental results are closer to each other with overlapping uncertainties. However, the CFD result is closer to the threshold than to the experiments. In addition, CI_E_ is overlapping with CI_Ω_ suggesting that the simulations do not provide sufficient validation at the prescribed level of significance (α = 0.05).

In addition to Eq 7, CI_E_ can also be obtained using U_val_ following the ASME V&V 20 standard as

$${\text{CI}}_{\text{E}}=\;\left[\text{E-}\left|{\text{U}}_{\text{val}}\right|{,\text{ E}+|\text{U}}_{\text{val}}|\right]$$ (8)

For this study, we evaluated both the threshold-based (Eq 7) and U_val_-based validation (Eq 8) approaches for the FDA nozzle model.

The threshold value for shear (in Eq 6) which varies as a function of residence time was obtained from previous studies [43], [45]. Raghunathan et al. [45] compiled and presented the threshold shear stress obtained from multiple studies for exposure times ranging from 10^−5^ s to 200 s. Depending upon the exposure time, the threshold shear for hemolysis varied from 100 Pa to 5000 Pa. The uncertainty values for the shear threshold (U_th_ in Eq 6), which are needed for the Student’s t-test statistics, were obtained from interpolating the data reported by Raghunathan et al.

## Results

The COU in this study is to evaluate the potential for hemolysis due to viscous shear stress in the nozzle geometry at Re = 3500 and 6500. Therefore, the results will focus on comparing and validating velocity and viscous shear at locations where the viscous shear stress is the greatest. The region where the quantitative comparison is made is marked in Fig 1b.

### Velocity vs. axial and radial distances

**Fig 3:**
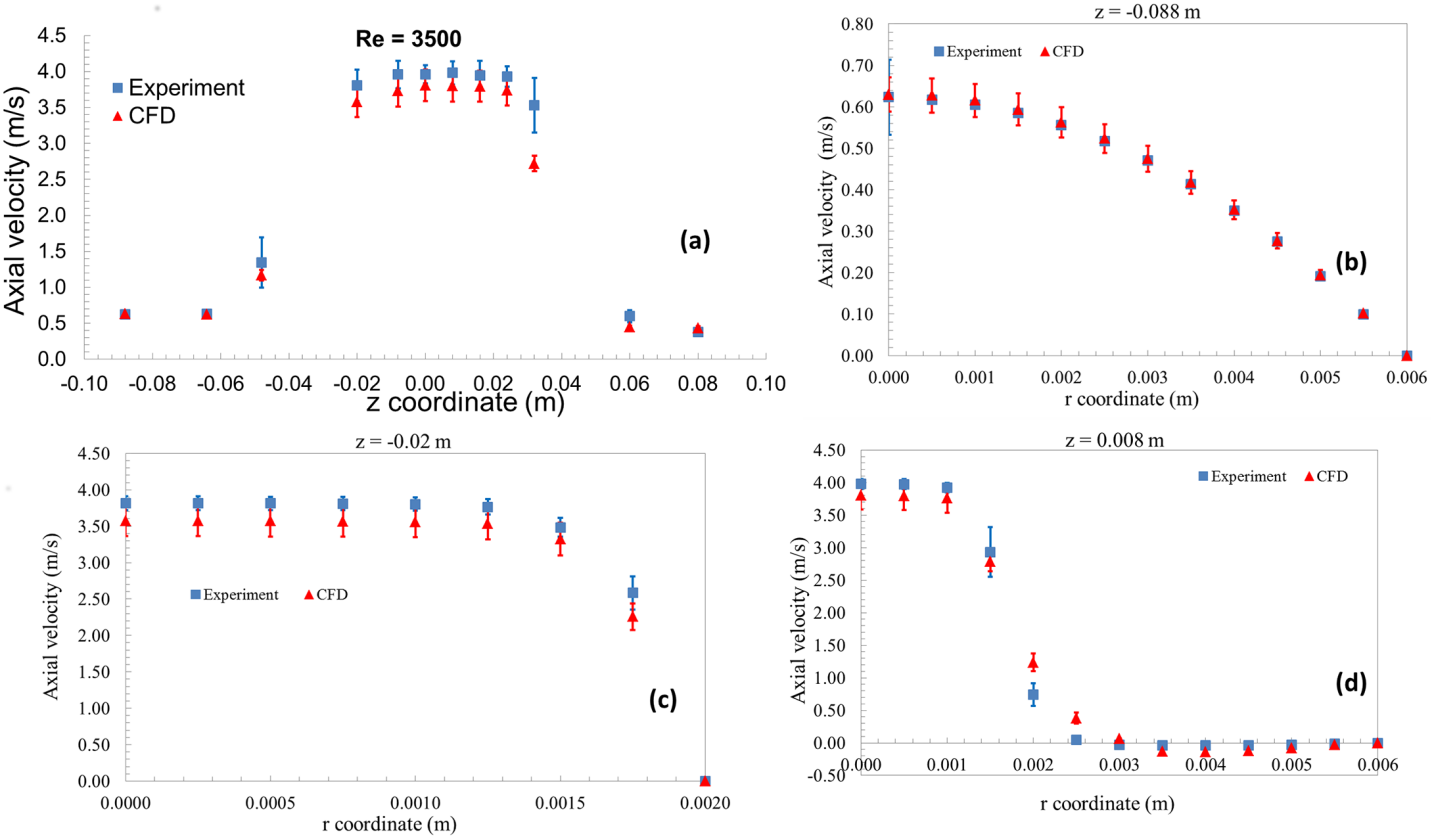
Velocity vs axial and radial distances with uncertainty bars for both CFD and PIV for Re = 3500.

**Fig 4:**
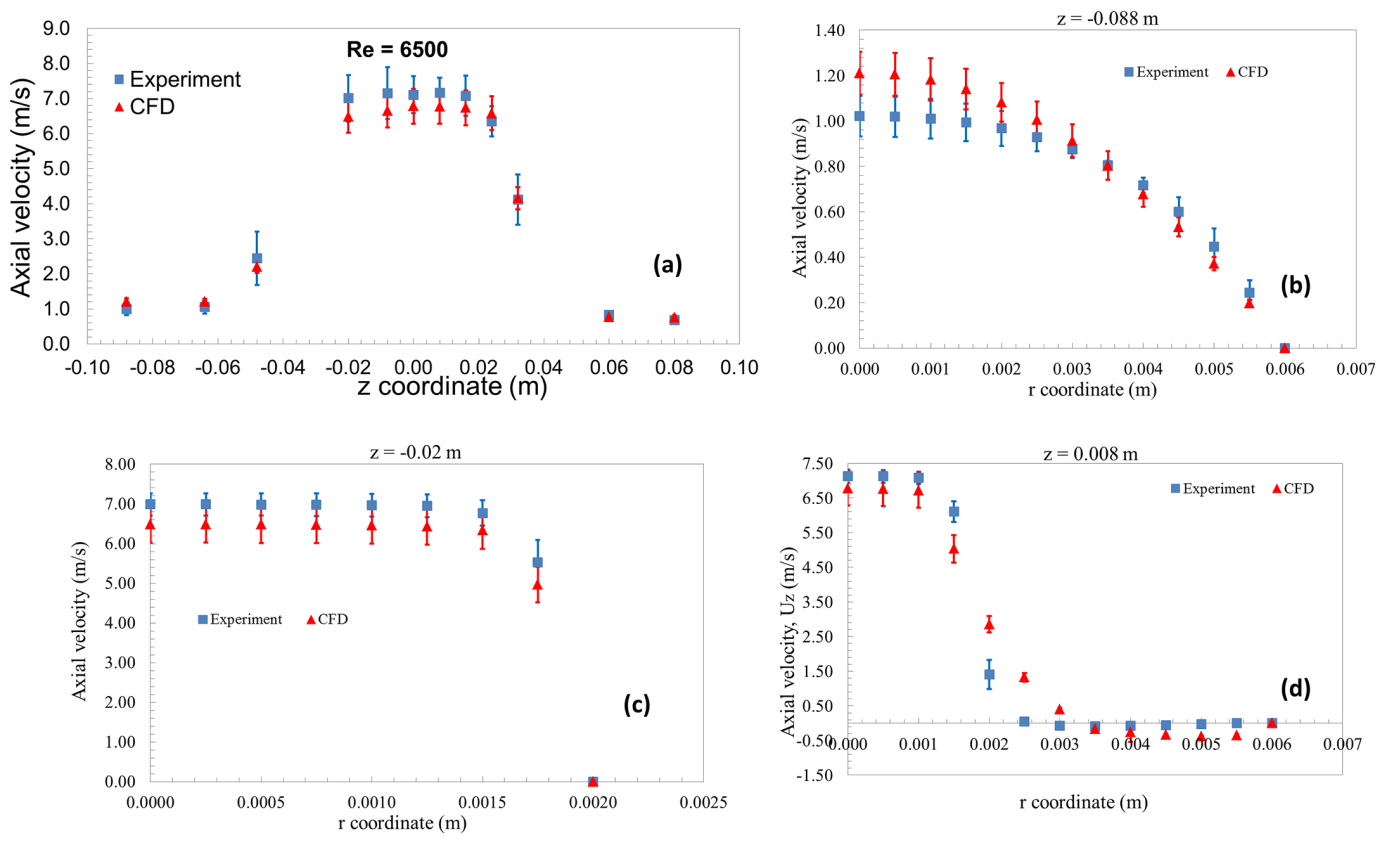
Velocity vs axial and radial distances with uncertainty bars for both CFD and PIV for Re = 6500.

Figs 3 and 4 compares the axial velocities from CFD and PIV as a function of axial (Figs 3a and 4a) and radial distances (Figs 3b–3d and 4b–4d) for Re = 3500 and 6500, respectively. The error bars accompanying the CFD data represent input parameter uncertainties while the experimental data includes the inter-laboratory variability. A qualitative comparison of the velocity data showed that the CFD results matched reasonably well with the experimental results at the entrance, throat, and sudden expansion of the nozzle geometry for both Re = 3500 and 6500 (Figs 3a and 4a). For Re = 3500, at the entrance and the throat regions, the mean velocity along the centerline and near the wall (Fig 3b and 3c) matched the experimental value within ~12% (Fig 3). For Re = 6500, the center-line velocity in the throat and the downstream regions matched within ~11%. Overall, the root mean square difference between the PIV and experiments for comparison locations (Fig 1b), normalized by the average velocity, was ~20% and ~15% for Re = 3500 and 6500, respectively. Of all the input parameters, flow rate is predicted to be the dominant source for uncertainty at the entrance with ~100% contribution, while the viscosity was the major contributor (~58%) at the reattachment region for Re = 3500.

### Shear stress vs. radial distance

**Fig 5:**
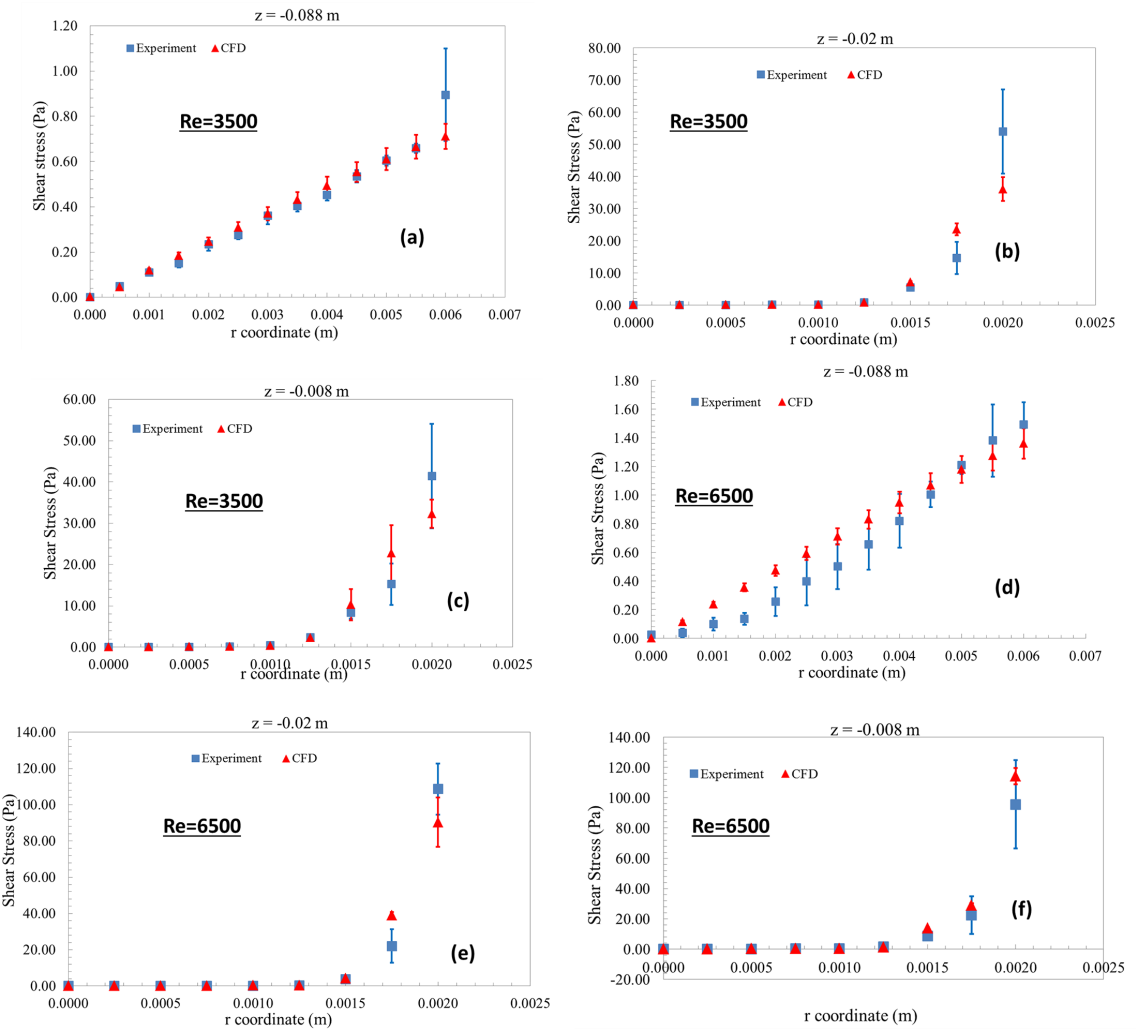
Shear stress vs radial distance with uncertainty bars for both CFD and PIV.

Fig 5 shows the shear stress data obtained at three different axial locations along the length of the nozzle. For the PIV experiments, the maximum shear stress (~55 Pa and 110 Pa for Re = 3500 and 6500, respectively) was observed in the throat region. Similar results were observed in the CFD simulations, with the peak viscous shear stresses estimated in the throat region of the model (Fig 5). However, the mean CFD data at the peak shear locations (Fig 5b and 5e) deviated from the experiment by ~33% and 20% for Re = 3500 and 6500, respectively.

### Validation

#### CFD vs PIV shear stress

**Fig 6:**
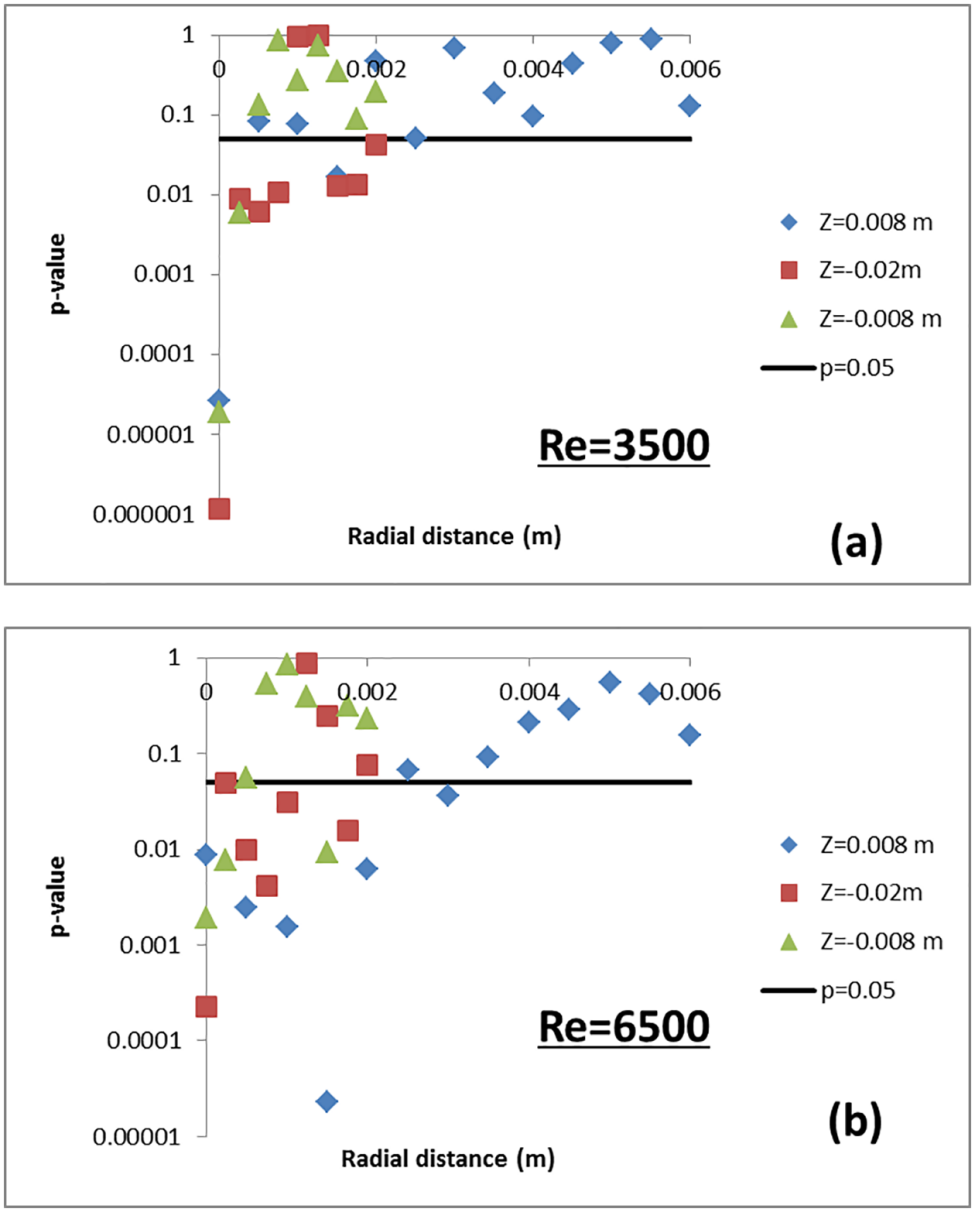
p-value vs radial distance at three axial locations i) entrance, z = 0.088m, ii) throat, z = -0.02 m, and iii) throat, z = -0.008 m. For reference, a straight line depicting p = 0.05 is shown in the figure. The p-values where obtained after performing a student’s t-test comparing the CFD and PIV shear stress datasets.

Having estimated the comparison error, numerical uncertainties and input-parameter uncertainties, the next step is to determine if the computational model can be considered sufficiently validated for the COU. To help with the validation process, a two-tailed Student’s t-test with unequal variance was performed to evaluate if the experimental and CFD datasets for shear stress, both represented by independent mean and standard deviation (Fig 5), were statistically different from one another. The standard deviation for the CFD and PIV shear-stress data is represented by U_sim_ and U_exp_, respectively. The CFD and PIV data were compared at three axial locations resulting in an independent p-value for each spatial location. In order to obtain the p-values, the t-test statistic is obtained for each spatial location as $\frac{\left|E\right|}{\sqrt{\left(S_{1}^{2}/n_{1}\right)+\left(S_{3}^{2}/n_{3}\right)}}$. The corresponding degrees of freedom is obtained as $\frac{{\left(S_{1}^{2}/n_{1}+S_{3}^{2}/n_{3}\right)}^{2}}{{\left(S_{1}^{2}/n_{1}\right)}^{2}/\left(n_{1}-1\right)+{\left(S_{3}^{2}/n_{3}\right)}^{2}/\left(n_{3}-1\right)}$, *n*_*1*_ and *n*_*3*_ are the number of samples associated with each variable (*n*_*1*_ = n3 = 5) and S_1_ = U_sim_ and S_3_ = U_exp_. Based on the t-test statistic and degrees of freedom, the corresponding p-values at each spatial location were computer using the standard TDIST function available in MS Excel (Microsoft Inc., Redmond, WA). Fig 6 shows the p-values obtained for the t-test as a function of radial distance for multiple axial locations. For reference, a straight line depicting p = 0.05 is drawn in the same figure. If the estimated p-values at different comparison locations are less than 0.05, then the CFD and PIV means are statistically different at a confidence level of 95%. The statistical comparison of the shear stress data obtained from CFD simulations and PIV measurements yielded p-values less than 0.05 at several locations (more for Re = 6500 (Fig 6b) than for Re = 3500 (Fig 6a)) in the throat and shear layer regions of the nozzle. These findings suggest that location-dependent differences exist between computational and experimental datasets and no general conclusions on model validity can be made based on direct comparison of shear stresses.

#### Threshold-based validation approach

**Fig 7:**
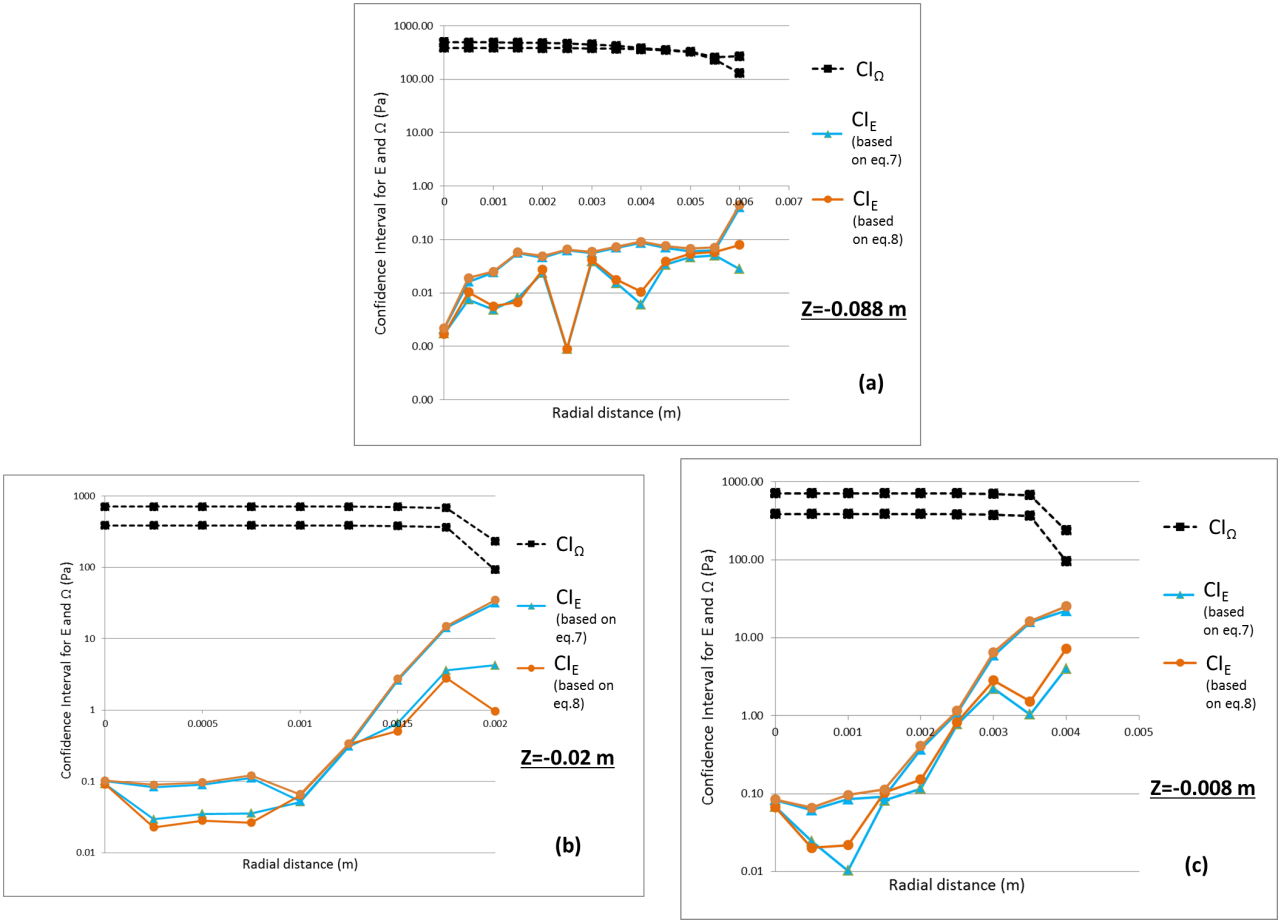
Confidence interval for |threshold-S| represented as CI_Ω_ and Error, E expressed as CI_E_ for a) entrance b) and c) throat for Re = 3500. Two lines for each CI represent the upper and lower bound values.

**Fig 8:**
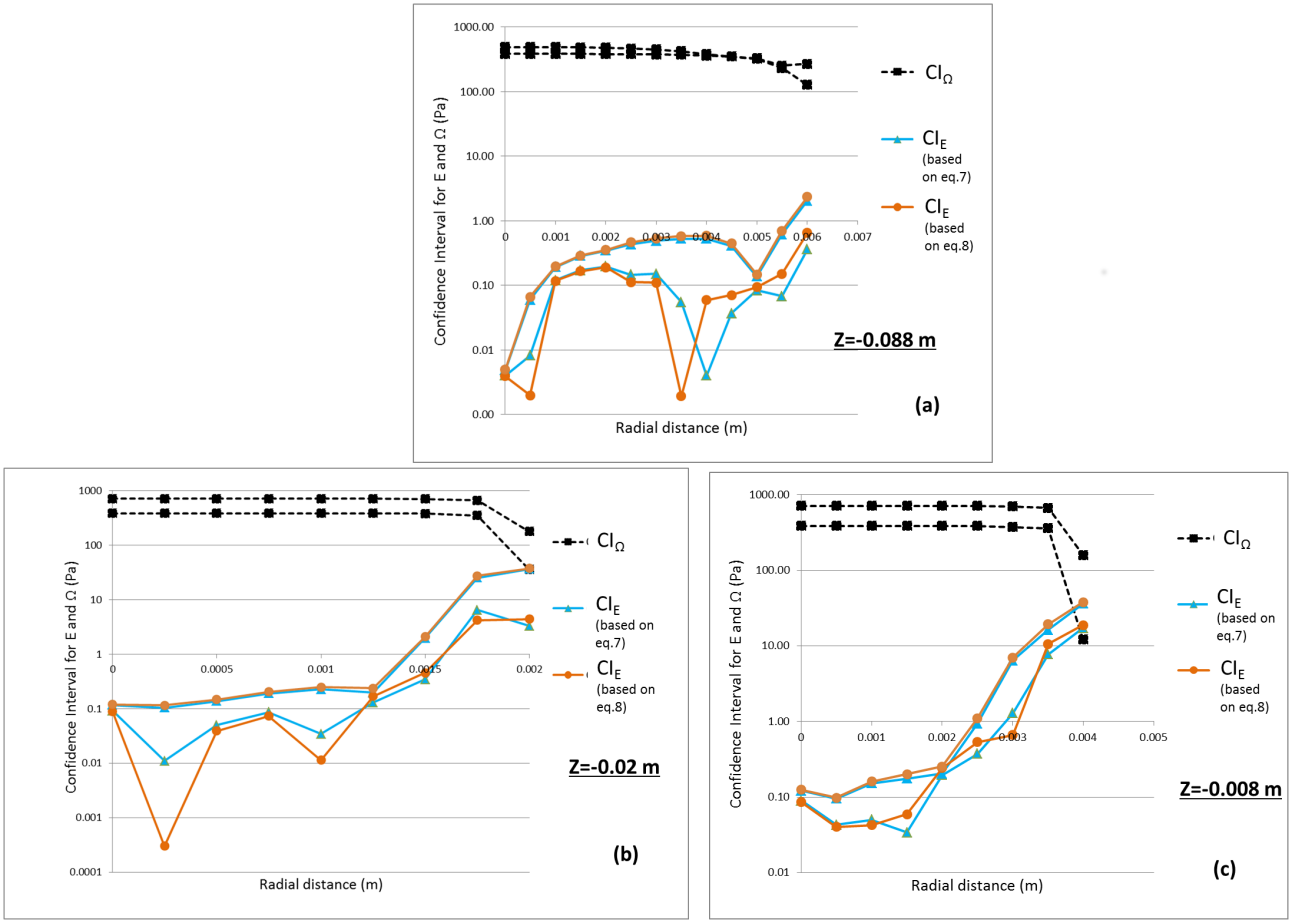
Confidence interval for |threshold-S| represented as CI_Ω_ and Error, E expressed as CI_E_ for a) entrance b) and c) throat for Re = 6500. Two lines for each CI represent the upper and lower bound values.

**Fig 9:**
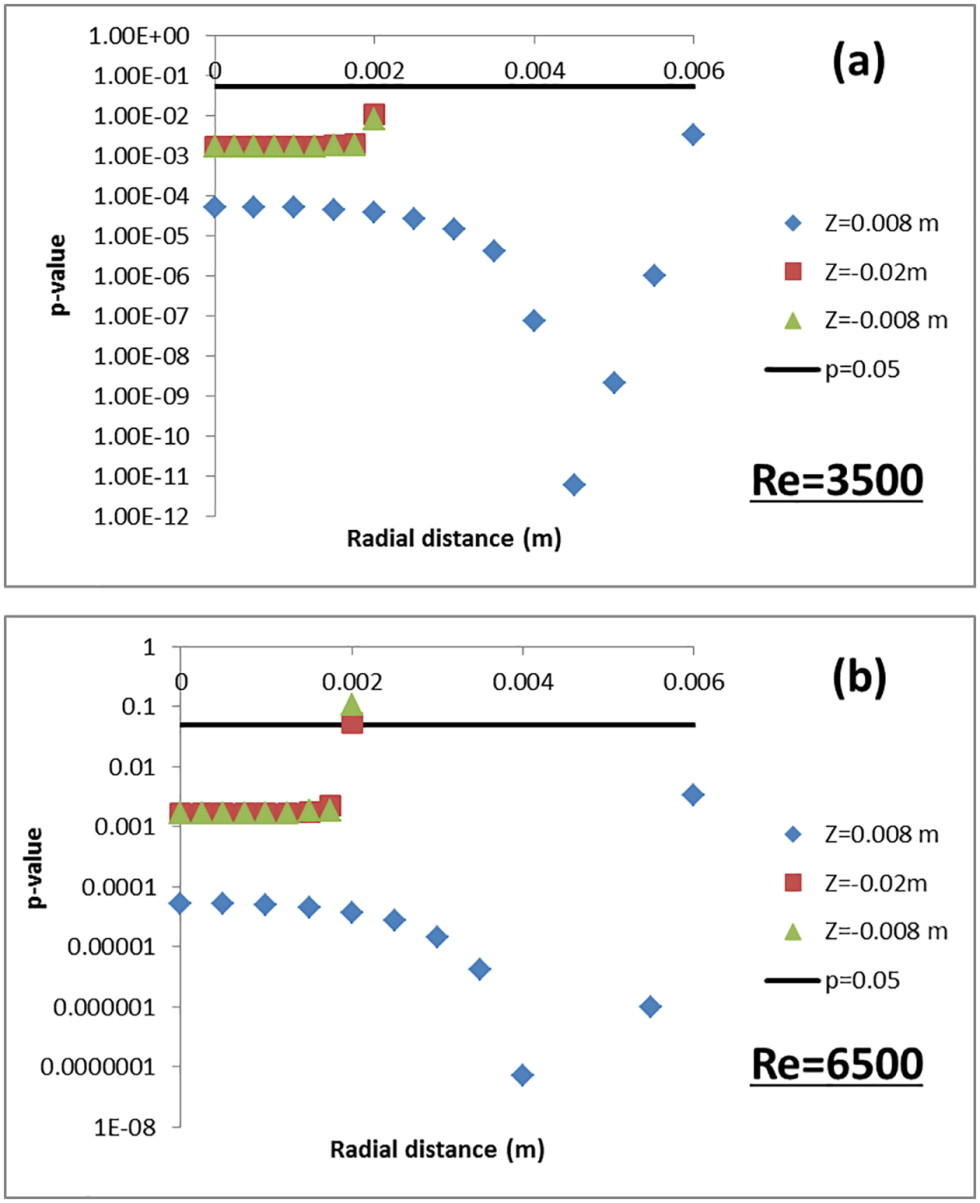
p-value vs radial distance at three axial locations i) z = 0.088m, ii) z = -0.02 m, and iii) z = -0.008 m for a) Re = 3500 and b) Re = 6500. For reference, a straight line depicting p = 0.05 is shown in the figure. The p-values where obtained after performing a Student’s t-test for Ω = |threshold—S| and E = |S-D|.

Figs 7 and 8 shows the confidence interval for *E* and *Ω* (estimated for shear stress) at the nozzle entrance and throat obtained from Eqs 6–8 for Re = 3500 and 6500, respectively. For all three locations, the CI_E_ was an order of magnitude below *CI*_*Ω*_ when Re = 3500 (Fig 7). Similar to Fig 6, another Student’s t-test was performed for E and *Ω* with the variance for the E and Ω represented as U_val_ and $\sqrt{U_{sim}^{2}+U_{th}^{2}}$, respectively. The p-values from the t-test are significantly less than 0.05, suggesting that the two datasets are statistically different from one another (Fig 9a). Stated another way, even when taking the simulation uncertainties into account, the shear stress predicted by the CFD model is extremely unlikely to exceed the threshold for blood damage; i.e. the potential for viscous shear-induced blood damage is unlikely at Re = 3500. On the contrary, for Re = 6500, the confidence interval for E overlapped with the CI_*Ω*_ especially at the peak shear locations (Fig 8). Accordingly, the p-values at these locations were above 0.05 (Fig 9b), and a conclusion that the shear stress predicted by the CFD model is below the threshold for blood damage cannot be made.

#### Sensitivity to the turbulence model

**Fig 10:**
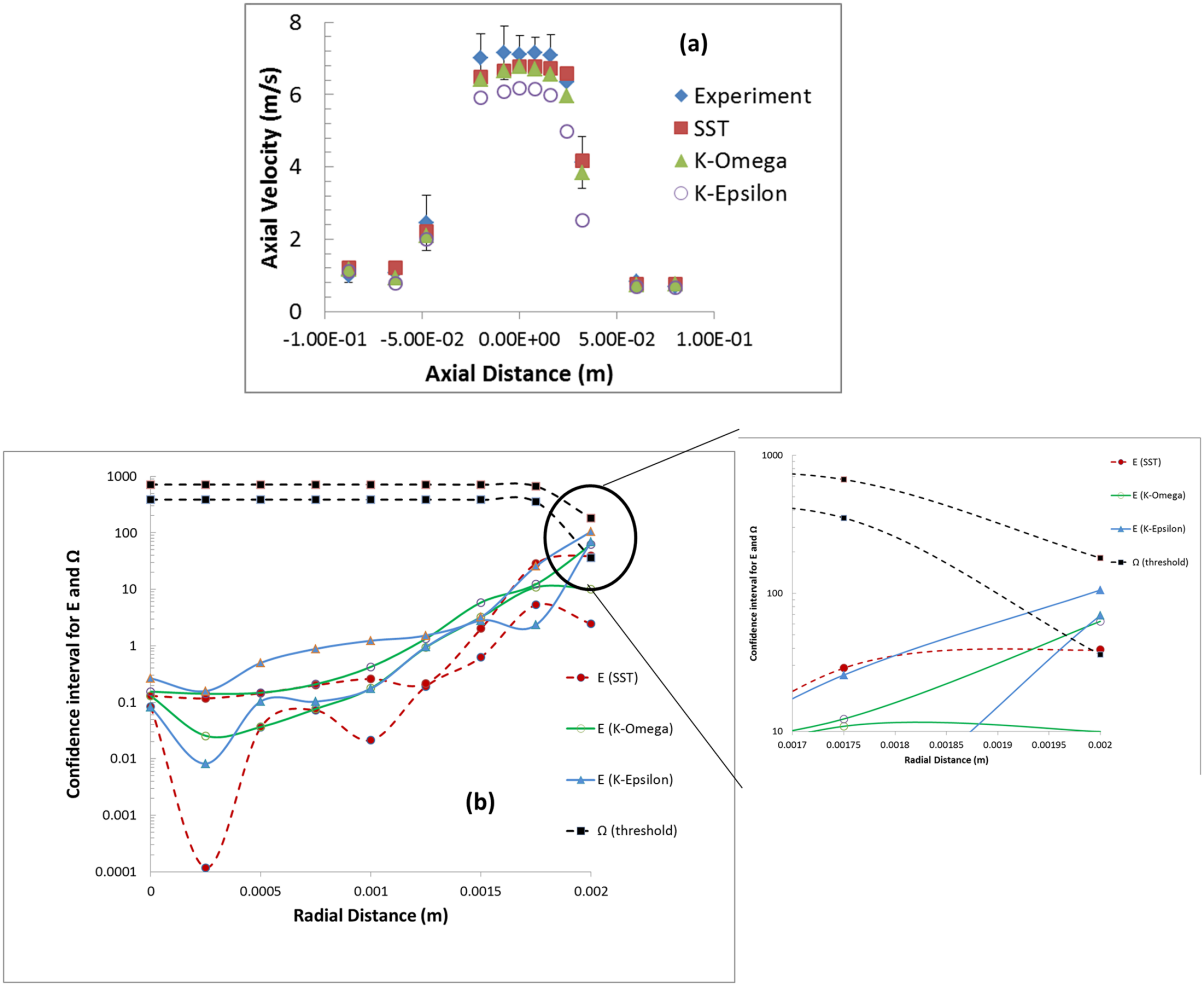
Sensitivity of center-line velocity (a) and Comparison error, E (b) to the choice of the turbulence model.

In order evaluate how the accuracy of the CFD results are affected by the choice of the turbulence model, additional simulations were performed at Re = 6500 for different Reynolds Averaged Navier Stokes (RANS) turbulence models. The RANS models used by the modelers during the FDA round-robin study of the nozzle geometry [33], namely SST k-ω, standard k-ω, and realizable k-ε turbulence models, were considered in this sensitivity study. The center-line velocity predicted in the throat and sudden expansion regions were within ~11%, 12%, and 39% of the mean experimental data for SST k-ω, k-ω, and k-ε models, respectively (Fig 10a). Fig 10b compares the Comparison error, E for all the three models with the proximity, Ω at one representative radial cross-section (z = -0.02m). The value for E at the peak-shear location was ~17%, 34%, and 80% for SST k-ω, k-ω, and k-ε models, respectively. The upper limit of E for SST k-ω and k-ω models overlapped slightly with the proximity. On the contrary, the confidence interval for K-ε overlapped entirely with the proximity interval. A similar trend was observed in other radial cross-sections in the throat and the sudden expansion regions.

## Discussion

The objective of this study is to develop a quantitative approach to evaluate the validity of a computational model in the presence of a known and measurable safety threshold. As an example, we chose the FDA nozzle model to determine if the instantaneous viscous shear stress is large enough to cause hemolysis at Re = 3500 and 6500.

The difference between experimental and computational measures of viscous shear stress was quantified statistically in terms of p-values derived from a t-test. The p-value increased with radial distance, partly due to the difficulty in making measurements near the wall.

Based solely on whether a statistically significant difference existed between experimental and CFD values of velocity and shear stress, the model could not be considered validated in certain regions (Fig 6). Consideration of the differences between PIV and CFD values might also lead to the conclusion that the model is more accurate at Re = 6500 than Re = 3500, because the RMS error obtained from all measurement locations was lower for Re = 6500 than Re = 3500. In addition, the peak shear predicted at the throat region (Fig 5) was more accurate for Re = 6500 (~17%) compared to Re = 3500 (~33% difference). However, for the purpose of predicting the likelihood of blood damage, the model is more useful at the Reynolds number of 3500, in the following sense.

For Re = 3500, our PIV and CFD results showed that the peak shear stress predicted by both techniques did not exceed 60 Pa, which is significantly below the shear stress threshold for red blood cell damage (> 100 Pa, [45]). Using our threshold-based approach, we were able to conclude that the difference between CFD and PIV is less than the difference between the CFD and the hemolysis threshold. In the throat region (Fig 7b), the upper limit of the 95% confidence interval for the comparison error, *CI*_*E*_ (for wall shear stress) is 3 times lower than the lower limit for the proximity to the threshold (*CI*_*Ω*_). In addition, the mean values for E are an order of magnitude less than Ω in the high shear regions (Fig 7b). A Student’s t-test performed between comparison error and the proximity to threshold also showed that the two datasets are statistically different (Fig 9a). In other words, the safety threshold is far enough away from the shear values encountered for this flow condition that the CFD model at Re = 3500 can be considered sufficiently validated based upon a safety-threshold criterion.

For Re = 6500, our threshold-based approach showed that the upper limit for E exceeds the lower limit for Ω in the throat region (Fig 8). Similarly, the p-values at the peak shear locations exceeded 0.05 suggesting that there is not enough evidence to indicate that the two datasets are statistically different. In other words, taking in to account the simulation, experimental, and threshold uncertainties, the CFD model cannot be considered sufficiently validated for predicting hemolysis arising from viscous shear stress at Re = 6500.

The lack of validation at Re = 6500 arises from the fact that, as threshold for safety is approached (Ω → 0), even if the mean CFD and PIV shear-stress are close (E≈0), the large U_th_, U_sim_, and U_exp_ values will cause the upper limit for E to exceed the lower limit for Ω. In order to adequately validate the CFD model at Re = 6500, the experimental, numerical and threshold uncertainties can be reduced. Alternatively, for the same level of uncertainty, the sample size can be increased. At the Reynolds number of 6500, if the level of uncertainty is maintained and the number of samples are increased, overlap between *CI*_*E*_ and *CI*_*Ω*_ no longer occurs when the number of samples exceeds 8, at the location z = -0.02 m. For close enough proximity to the damage threshold (Ω ≈ 0), it becomes impractical to reduce the uncertainty further, or increase the sample size, and the model cannot be validated at a sufficiently high level to credibly predict damage in this regime. If it is desired to continue to operate the device extremely close to the damage threshold, blood-damage predictions can be derived from methods other than computational models, e.g. in-vivo experiments. The strength of our threshold approach is that it imposes a more stringent validation acceptance criterion as the CFD shear-stress approaches the safety threshold. This enhanced scrutiny is warranted since the risk to patient safety increases as the threshold is approached. On the other hand, for Reynolds numbers significantly greater than 6500, as the CFD shear stresses become significantly larger than the threshold value, the validation criteria will become less stringent again. However, for large Reynold numbers (>6500), even if the model is sufficiently validated the device will be deemed hemolysis-prone.

The uncertainty estimates derived in the paper constitute a measure of credibility of the validation process. The uncertainty limits for the computational data (Fig 5) are not just an indicator of the model accuracy, but also for how well the experimental input parameters were controlled and numerical errors in the simulations were minimized. Consequently, the information from the uncertainty study can be used to identify the key input parameters that affect the accuracy of the model output. Subsequently, the validation process can be made more credible by improving the experimental protocol and minimizing the uncertainties in the key input parameters.

We also evaluated how the uncertainties in the CFD data were influenced by the choice of the turbulence model. Our results showed that of all the models used in the FDA round-robin study [33], SST k-ω was the closest to the experimental results. In the future, this sensitivity study should be expanded to include more turbulence models including large eddy simulation (LES) [36]. For each turbulence model, the U_val_ and E values need be calculated again since the uncertainties in the variable of interest will vary with the choice of the turbulence model used in the CFD simulations.

It should be noted that the CFD model was validated with the experimental data only at Re = 3500. Because the validation was performed for the turbulent flow regime, additional validation experiments are needed before the model can be used for a broader COU. For example, if one additional validation experiment was performed at Re = 1500 (assuming laminar flow was observed experimentally at this Re), then the laminar CFD model would be applicable to all Reynolds number below 1500. This is possible because the viscous shear stress values in the throat region will decrease with the Reynolds number and the difference between *CI*_*E*_ and the *CI*_*Ω*_ will be wider than what was estimated at Re = 1500. The turbulent CFD model could be extended for Reynolds number greater than 6500 as long as the modelers show that the comparison error proportionally scales with the Reynolds number and that *CI*_*E*_ remains non-overlapping with *CI*_*Ω*_. Similarly, the model can also be used for different flow geometries for comparable flow rates, without further validation, as long as the flow field created in the new flow geometry is similar to the validation model.

Any significant change in the question of interest or the COU requires a new set of validation metrics or even new validation thresholds. For example, because the model was not able to accurately predict the reattachment location and reattachment length for Re = 3500 (Fig 3a), the model may not be valid for evaluating the risk of platelet aggregation and thrombosis initiation in the device, unless the predicted values are sufficiently far from the established thresholds where undesired effects occur. Such a determination would require a threshold appropriate for those bioeffects. Similarly, for Re = 6500, the current COU focused on determining if the CFD model is valid to estimate if instantaneous viscous shear can cause localized hemolysis in the nozzle. However, the COU could be changed to evaluate the total amount of hemolysis caused by the device at this Reynolds number. Under such a scenario, the validation experiments should involve measuring the overall hemolysis caused by the device in addition to measuring the shear stress. The CFD model will also involve two parts i) fluid flow modeling to determine the velocity and shear stress and ii)hemolysis modeling to predict the free-hemoglobin concentration. Since the shear-stress exceeded the threshold only in few spots on the nozzle geometry, the total amount of free hemoglobin released from the nozzle could potentially be negligible [31]. Consequently, the CFD model could be validated for Re = 6500 by changing the COU and comparing with in vitro hemolysis results.

Our study showed that the threshold-based approach is applicable in situations where the experimental and input parameter uncertainties are relatively large for the variable of interest (Figs 3 and 4). Such situations are common during the testing of implantable medical devices where accurate characterization of boundary conditions is often difficult to perform, creating situations where modelers have to manage large uncertainties in input parameters. It should be noted that other forms of evidence used for evaluating device safety such as animal testing or clinical trials carry potentially larger uncertainties compared to the simulation results. So, it is critical to use all forms of evidence in a collective manner while evaluating the safety of medical devices. The validation criteria developed following the threshold approach will not only be a function of Comparison error, E, but also takes in to account the risk to patient safety because of E. We believe that such a validation approach has the potential to enhance the credibility and influence of computational modeling in the medical device regulatory process.

Our threshold-based validation approach works well for scenarios where the modeler is able to thoroughly quantify the validation uncertainty and differentiate the model-form error and the uncertainties from experiments, input parameters, and numerical discretization. In order to quantify the numerical and input parameter uncertainties, we used the sensitivity coefficient method prescribed by the ASME V&V 20–2009 standard. The sensitivity method was used for illustrative purposes, but other well-established uncertainty quantification methods could be used in lieu of the sensitivity coefficient approach. The sensitivity coefficient method approach was justified since our goal was to obtain the uncertainty values for well-defined flow conditions (Re = 3500 or 6500) and the uncertainties in the most sensitive parameters (flow rate and viscosity) occupied only a small neighborhood (less than 8%) around the nominal value. The main drawback of this local approach is that it assumes a linear relation as the parameter range is expanded and will not capture any non-linear behavior of the system response. The ASME V & V 20–2009 standard also outlines a Monte-Carlo method for global uncertainty quantification. In addition, as discussed in the Introduction, several researchers have proposed other types of global approaches for estimating the input parameter uncertainties [23–26, 29]. These alternate uncertainty quantification techniques, should also be compatible with the threshold-based validation approach.

It should be noted that the existing V&V standards [14, 15], while providing a detailed methodology for quantifying the comparison error, do not provide a quantitative approach for deciding when a computational model is considered validated or credible. Such a determination is left to the practitioner’s judgement. However, our study describes an approach and provides quantitative methodology for evaluating the validity of a proposed computational model when a threshold value is available for evaluating device safety.

The subjective part of our threshold-based validation approach is the selection of appropriate ‘p-value’ from the Student’s t-test for estimating the confidence interval for the comparison error. For this study, we selected a p-value of 0.05 (95% confidence interval). Other p-values can be selected, depending in part on the model risk. Model risk, as defined by the (draft) ASME V & V 40 standard, is a combination of two factors: the influence of the computational model on the decision-making process, and the consequence of that decision on the patient or other non-patient related impacts. For situations where the model risk is high (high influence and high consequence) more conservative values for the validation criteria (‘p-value’) might be selected. In addition, a safety factor (SF) could be used to reduce the proximity value (Ω*1/SF) and ensure that the confidence intervals for *CI*_*Ω*_ and *CI*_*E*_ are far apart from one another. On the contrary, less stringent validation criteria might be selected when the model risk is low, such as in situations where the computational model has negligible or minor influence in the decision making process, and/or the safety claims are mainly based on significant contributions from other sources of data.

The credibility of the threshold-based validation method is dependent on the accuracy of the threshold value. In general, the safety thresholds are not obtained directly from the validation experiments or CFD simulations but from prior experiments and published literature. Consequently, including the uncertainty values for the threshold (and not just for experiments and simulations) is required for credibility of the validation process. To derive the uncertainty in the threshold, the variability between different studies of the threshold can be used, as in our approach. If only a single threshold study is available, the measurement uncertainty for the study can be used for the threshold uncertainty, perhaps magnified using a factor of safety.

**Fig 11:**
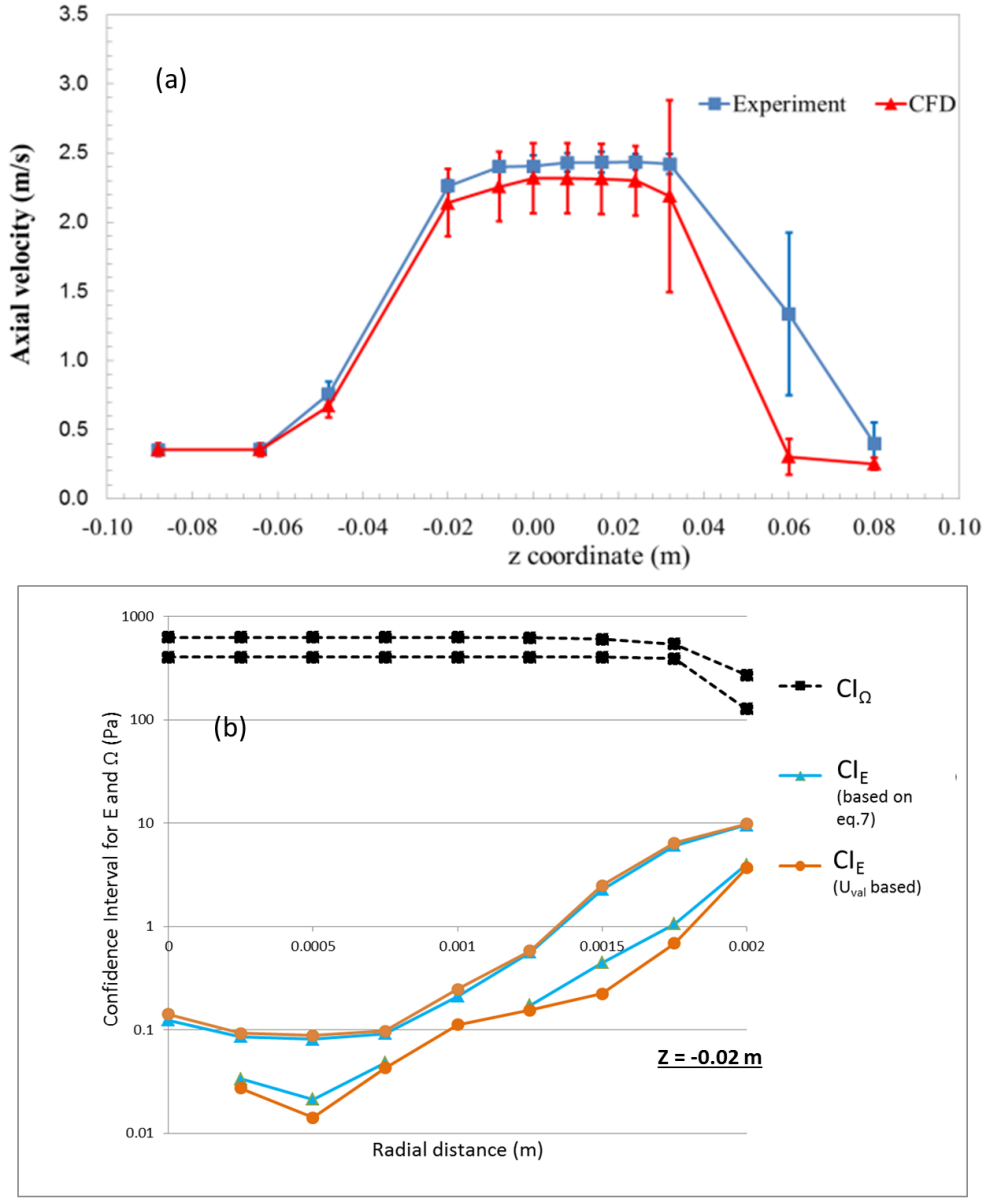
a) Centerline velocity vs axial distance at Re = 2000, b) Confidence interval for |threshold-S| represented as CI_Ω_ and Error, E expressed as CI_E_ at the throat for Re = 2000. Two lines for each CI represent the upper and lower bound values.

While applying the threshold approach, there is a risk of validating an inadequate model because the threshold is far away from the values predicted by the simulations. For example, Fig 11 shows the center-line velocity and the confidence intervals for E and Ω for the nozzle geometry at Re = 2000. By comparing E and Ω, one could conclude that the SST k-ω model is valid enough to predict shear stress at transitional flow regime (Re = 2000). However, a closer evaluation of the center-line velocity shows that the simulations results deviate from the experiments by as much as 100%. The excessive dissipation introduced by the RANS turbulent model was causing premature jet reattachment in the sudden expansion region when compared to the experiments. Therefore, the use of a RANS turbulence model may not be appropriate in the transitional flow regime and an LES model might be more appropriate at Re = 2000. However, applying the threshold approach before evaluating the velocity data could lead to the conclusion the RANS turbulence mode is valid to make viscous shear predictions at Re = 2000. Consequently, before applying the threshold approach, the modelers should ensure that the model used in the simulation represents the physics of interest as closely as possible. This can be achieved by evaluating the sensitivity of the simulations results to different model form (RANS vs laminar vs LES). We performed a similar analysis by comparing different RANS models at Re = 6500 (Fig 10). Alternatively, the modelers could also use an acceptance criterion that defines the level of agreement between the CFD and experimental results before applying the threshold data. This acceptance criterion could also be related the model risk with the high risk models requiring a more stringent acceptance criterion for E. In addition, before applying the threshold approach, the modelers should ensure that all the best practices for CFD simulations are followed such as mass conservation, adequate mesh quality, and higher order of accuracy. All these additional precautions will ensure that the threshold approach is used in more meaningful manner for making device safety or effectiveness claims.

In conclusion, this study applied the V & V methodology published by the ASME V&V 20–2009 standard on the FDA nozzle model to understand and highlight the need for quantifying the experimental and numerical uncertainties as a part of the validation process. This study also extended the methodology and provided a strategy for determining if the model can be deemed sufficiently validated for a given COU in the presence of clear safety thresholds. The ASME V &V 20–2009 standard and the threshold-based validation approach can be used to enhance the credibility and influence of computational modeling in the safety evaluation of all types of medical devices.

## Nomenclature

*S*: CFD simulation results
*D*: Experimental data
*E*: Comparison error
*Threshold*: Viscous shear-stress safety threshold for hemolysis which is a function of residence time
*Ω*: Proximity, Magnitude of difference between simulation and threshold = |Threshold-S|
*U*_*val*_: Validation uncertainty
*Re*: Reynolds number in the nozzle throat
*Q*: Inlet flow rate, m^3^/s
*d*: Throat diameter, m
*μ*: Mean dynamic viscosity, Pa.s
*ρ*: Mean density, kg/m^3^
${\bar{U}}_{z}$
Average axial velocity, m/s
*R*: Nozzle inlet radius, m
*r*: Radial co-ordinate
*z*: Axial co-ordinate
*TI*: Turbulent intensity (%)
*U*_*num*_: Numerical uncertainty
*h*: grid size
Δ*V*_*i*_: Volume of the i^th^ grid element
*N*: Total number of grid elements
*w*: Grid refinement factor
*F*_*s*_: Factor of safety for grid refinement study
*φ*: Output variable of interest (velocity or shear stress) in the grid refinement study
*ε*: Absolute error in the output variable of interest, *φ*
*θ*: Order of convergence in the grid refinement study
*τ*: Shear stress, Pa
*τ*_*w*_: Wall shear stress, Pa
*U*_*inp*_: Uncertainty in simulation output (velocity or shear stress) due to uncertainty in input parameters such as Q, μ, and TI
*X*_*i*_: ith input parameter in the sensitivity coefficient method
$\frac{\partial S}{\partial X_{i}}$
Sensitivity coefficient for simulation output parameter S
$u_{x_{i}}$
Experimental uncertainty in the ith input parameter for the sensitivity coefficient method
Δ*X*_*i*_: Change in the ith input parameter for the sensitivity coefficient method
*U*_*exp*_: Uncertainty in the experiment output data (velocity or shear stress)
*δ*_*model*_: Modeling error which is the error in the simulation due to an incorrect choice of the model
*U*_*th*_: Uncertainty in the shear stress threshold which is also a function of residence time
*CI*_*Ω*_: Confidence interval for Ω
*CI*_*E*_: Confidence interval for Comparison error, E
*d”*: Degree of freedom for the Student’s t-test
*B*_*1*_: Variance for CFD, dataset for the Student’s t-test
*B*_*2*_: Variance for threshold for the Student’s t-test
*B*_*3*_: Variance for PIV dataset for the Student’s t-test
